# Fitness Impact of Obligate Intranuclear Bacterial Symbionts Depends on Host Growth Phase

**DOI:** 10.3389/fmicb.2016.02084

**Published:** 2016-12-22

**Authors:** Chiara Bella, Lars Koehler, Katrin Grosser, Thomas U. Berendonk, Giulio Petroni, Martina Schrallhammer

**Affiliations:** ^1^Microbiology, Institute of Biology II, Albert-Ludwigs Universität FreiburgFreiburg, Germany; ^2^Zoology-Anthropology Unit, Biology Department, Università di PisaPisa, Italy; ^3^Institute of Hydrobiology, Technische Universität DresdenDresden, Germany

**Keywords:** *Holospora caryophila*, *Paramecium biaurelia*, fitness reduction, context-dependent mutualism, endosymbiont, parasite, beneficial effect, symbiosis

## Abstract

According to text book definition, parasites reduce the fitness of their hosts whereas mutualists provide benefits. But biotic and abiotic factors influence symbiotic interactions, thus under certain circumstances parasites can provide benefits and mutualists can harm their host. Here we addressed the question which intrinsic biotic factors shape a symbiosis and are crucial for the outcome of the interaction between the obligate intranuclear bacterium *Holospora caryophila* (*Alphaproteobacteria; Rickettsiales*) and its unicellular eukaryotic host *Paramecium biaurelia* (Alveolata; Ciliophora). The virulence of *H. caryophila*, i.e., the negative fitness effect on host division and cell number, was determined by growth assays of several *P. biaurelia* strains. The performances of genetically identical lines either infected with *H. caryophila* or symbiont-free were compared. Following factors were considered as potentially influencing the outcome of the interaction: (1) host strain, (2) parasite strain, and (3) growth phases of the host. All three factors revealed a strong effect on the symbiosis. In presence of *H. caryophila*, the *Paramecium* density in the stationary growth phase decreased. Conversely, a positive effect of the bacteria during the exponential phase was observed for several host × parasite combinations resulting in an increased growth rate of infected *P. biaurelia*. Furthermore, the fitness impact of the tested endosymbionts on different *P. biaurelia* lines was not only dependent on one of the two involved strains but distinct for the specific combination. Depending on the current host growth phase, the presence of *H. caryophila* can be harmful or advantageous for *P. biaurelia*. Thus, under the tested experimental conditions, the symbionts can switch from the provision of benefits to the exploitation of host resources within the same host population and a time-span of less than 6 days.

## Introduction

The concept of symbiosis (Oulhen et al., 2016) comprises mutualism and parasitism. According to biological textbooks (e.g., Reece et al., 2014), mutualists provide benefits to their host whereas parasites explore its resources and hence cause a fitness reduction. Despite this unambiguous definition, in many biological systems we do not find a neat separation between mutualism and parasitism. Several studies revealed that environmental factors can influence symbiotic interactions and, under certain circumstances, parasites can provide benefits and mutualists can harm their host (reviewed by Leung and Poulin, 2008; Pérez-Brocal et al., 2013).

Examples for bacterial parasites are *Holospora* (*Alphaproteobacteria;* Amann et al., 1991) and *Holospora-*like bacteria (Boscaro et al., 2013), intranuclear symbionts of the ciliate *Paramecium* (Alveolata; Ciliophora). These bacteria possess a life cycle with two alternate morphologies: a short, reproductive (RF) and a longer, infectious (IF) form (Görtz et al., 1989; Fujishima et al., 1990; Fokin et al., 1996). IF are agents of horizontal transmission. Infection of new *Paramecium* hosts occurs after phagocytosis and escape of the IF from the phagosome. Once the IF have reached their target nucleus via actin-based motility (Sabaneyeva et al., 2009), they differentiate into RF. Those multiply by binary division and ultimately develop back to IF. During host cell division, RF are vertically transmitted to the nuclei of the daughter cells and IF are released into the medium for a new round of horizontal transmission (Görtz and Schmidt, 2005; Fujishima, 2009).

Living in the nuclear compartment, these bacteria have adapted to the particular nutrient availability of their habitat. An ATP/ADP translocase was described from *Holospora obtusa* (Linka et al., 2003). This protein belongs to a class of nucleotide transporters present in several obligate intracellular bacteria such as the human pathogens *Rickettsia* and *Chlamydia* (Winkler and Neuhaus, 1999; Amiri et al., 2003) and several protozoan endosymbionts, i.e., *Caedibacter caryophilus* (Daugherty et al., 2004), *Protochlamydia amoebophila* (Schmitz-Esser et al., 2004; Haferkamp et al., 2006), and, as already mentioned, *Holospora obtusa*. It enables the bacteria to actively take-up ATP from the host cell. Therefore, these endosymbionts have been termed “energy parasites” (Hatch et al., 1982; Schulz and Horn, 2015). *Holospora undulata* for instance causes a reduction of *Paramecium caudatum per capita* growth rate and carrying capacity (Banerji et al., 2015).

In this study, the effect of the intranuclear symbiont *Holospora caryophila* on *Paramecium biaurelia* was examined. The susceptibilities of naïve *P. biaurelia* exposed to isolated bacteria were assessed at different stages of the infection process. Additionally, virulence of the symbionts or in other words reduction of their host's fitness was determined by performing growth assays with genetically identical *P. biaurelia* lines either harboring or lacking *H. caryophila*. Considering strain identity of (1) host, (2) parasite, and (3) the host growth phase, a strong influence of all three factors on the outcome of the interaction was observed during infection as well as in the established symbiosis. In many cases infected paramecia revealed a decreased density in the stationary phase but a stronger increase during exponential growth when compared to the respective symbiont-free line.

## Materials and methods

### *Paramecium* lines and cultivation

Paramecia in four different infection states were used in this study, i.e., chronically and experimentally infected, naïve, and cured cells (Table 1). All cultures were kept at 20°C in semi-batch cultivation in 0.25% Cerophyll medium (CM; 0.25% wheat grass (GSE-Vertrieb, Saarbrücken, Germany), 398.75 mg l^−1^ Na_2_HPO_4_, 135.5 mg l^−1^ NaH_2_PO_4_, 104 mg l^−1^ NaCl, 40 mg l^−1^ MgSO_4_, 85 mg l^−1^ MgCl_2_, 13.5 mg l^−1^ CaCl_2_, 23 mg l^−1^ KCl, 500 μg l^−1^ Stigmasterol (Sigma-Aldrich, Munich, Germany). Once a week 1/10 to 1/4 of the culture volume was replaced with bacterized CM (0.25% CM inoculated with *Raoultella planticola* DMSZ 3069 as food organism 2 days before use and incubated at 20°C).

**Table 1:** **Origin and infection status of *Paramecium biaurelia* lines**.

***P. biaurelia* line**	**Infection status**	**Used in fitness assay**	**Isolated/Generated by**	**Geographic origin**
FGC3_chronic	Chronically infected with *H. caryophila* FGC3	Yes	S. Galati	Calabria, Italy
FGC3_AB	Cured (line derives from *P. biaurelia* FGC3)	Yes	This study	−
GFg_chronic^a^	Chronically infected with *H. caryophila* GFg	Yes	Castelli et al., 2015	Freiburg, Germany
GFg_AB^a^	Cured (line derives from *P. octaurelia* GFg)	Yes	This study	−
Hc^+^_chronic	Chronically infected with *H. caryophila* Hc^+^	No	S. Fokin	Münster, Germany
Hc^+^_AB	Cured (line derives from *P. biaurelia* Hc^+^)	No	This study	−
562α_chronic	Chronically infected with *H. caryophila* 562α	Yes	Beale et al., 1969	Milan, Italy
562α_AB	Cured (line derives from *P. biaurelia* 562α)	Yes	This study	−
Anti	Naïve	Yes	Potekhin et al., 2010	Antibes, Siberia, Russia
Dub	Naïve	Yes	Potekhin et al., 2008	Dubna, Moscow, Russia
Opa	Naïve	Yes	Przyboś et al., 2011	Opatowice, Krakow, Poland
Ri	Naïve	Yes	Potekhin et al., 2010	Rieff, Scotland, Great Britain
Yama	Naïve	Yes	Przyboś and Surmacz, 2010	Yamaguchi, Yuu, Japan
Rybi	Naïve	No	Potekhin et al., 2010	Rybinskoye, Yaroslavl, Russia
Tas	Naïve	No	Przyboś and Surmacz, 2010	Tasmania, Australia
Kra	Naïve	No	Komala and Przyboś, 1999	Kraków, Poland
Anti_FGC3	Experimentally infected with *H. caryophila* FGC3	Yes	This study	−
Dub_FGC3	Experimentally infected with *H. caryophila* FGC3	Yes	This study	−
Opa_FGC3	Experimentally infected with *H. caryophila* FGC3	Yes	This study	−
Ri_FGC3	Experimentally infected with *H. caryophila* FGC3	Yes	This study	−
Yama_FGC3	Experimentally infected with *H. caryophila* FGC3	Yes	This study	−
Rybi_FGC3	Experimentally infected with *H. caryophila* FGC3	No	This study	−
Anti_Hc^+^	Experimentally infected with *H. caryophila* Hc^+^	Yes	This study	−
Dub_Hc^+^	Experimentally infected with *H. caryophila* Hc^+^	Yes	This study	−
Opa_Hc^+^	Experimentally infected with *H. caryophila* Hc^+^	Yes	This study	−
Ri_Hc^+^	Experimentally infected with *H. caryophila* Hc^+^	Yes	This study	−
Yama_Hc^+^	Experimentally infected with *H. caryophila* Hc^+^	No	This study	−
Rybi_Hc^+^	Experimentally infected with *H. caryophila* Hc^+^	No	This study	−
Anti_562α	Experimentally infected with *H. caryophila* 562α	No	This study	−
Dub_562α	Experimentally infected with *H. caryophila* 562α	No	This study	−
Opa_562α	Experimentally infected with *H. caryophila* 562α	Yes	This study	−
Ri_562α	Experimentally infected with *H. caryophila* 562α	No	This study	−
Yama_562α	Experimentally infected with *H. caryophila* 562α	Yes	This study	−
Rybi_562α	Experimentally infected with *H. caryophila* 562α	No	This study	−
Anti_GFg	Experimentally infected with *H. caryophila* GFg	No	This study	−
Dub_GFg	Experimentally infected with *H. caryophila* GFg	No	This study	−
Opa_GFg	Experimentally infected with *H. caryophila* GFg	No	This study	−
Ri_GFg	Experimentally infected with *H. caryophila* GFg	No	This study	−
Yama_GFg	Experimentally infected with *H. caryophila* GFg	No	This study	−
Rybi_GFg	Experimentally infected with *H. caryophila* GFg	No	This study	−

a *The host does not belong to the species P. biaurelia, but to the closely related Paramecium octaurelia*.

*P. biaurelia* strains 562α, FGC3, Hc^+^ (carrying *H. caryophila* 562α, FGC3, or Hc^+^, respectively), and *Paramecium octaurelia* GFg (with *H. caryophila* GFg) were used as chronically infected lines (naturally infected since more than 7 years). Eight strains of *P. biaurelia* were considered naïve (previously not exposed to infection, at least since isolation). The four chronically infected strains were additionally cured (cured lines) from their endosymbionts by antibiotic treatment. Experimentally infected lines were generated by infection of the naïve *Paramecium* strains with *H. caryophila* 562α, FGC3, Hc^+^, or GFg, respectively in the described and additional infection experiments (data not shown).

Selection criterion for lines used in the fitness assay was a high infection prevalence in the range from 82 to 100% as determined at the beginning and at the end of the experiment. In total 20 *P. biaurelia* lines (Table 1) belonging to the four above mentioned infection states were examined in the assay.

### Antibiotic treatment

Chronically infected cells of *P. biaurelia* 562α, FGC3, Hc^+^, and *P. octaurelia* GFg were cured from *H. caryophila*. Therefore, individual cells were washed three times and incubated for 24 h in sterile Volvic mineral water (Danone Waters GmbH, Germany) containing 250 μg ml^−1^ streptomycin (Sigma, St. Louis, USA). The next day, bacterized medium was added and cells were allowed to grow until a sufficient cell density was reached to control the infection status with fluorescence *in situ* hybridization (FISH). If necessary (when *H. caryophila* was still observed), a repeated treatment (as described above) in an antibiotic solution containing 125 μg ml^−1^ streptomycin and 125 μg ml^−1^ chloramphenicol (Sigma, St. Louis, USA) was performed.

### Experimental infection

Naïve paramecia were experimentally infected by *H. caryophila* isolated from chronically infected strains. Therefore, donor cells were harvested by centrifugation (1200 rpm, 20 min, centrifuge K 26 D, MLW, GDR) and washed in 1x PBS (137 mM l^−1^ NaCl, 2.7 mM l^−1^ KCl, 10 mM l^−1^ Na_2_HPO_4_, 1.8 mM l^−1^ KH_2_PO_4_). The concentrated cells were mechanically lysed by vortexing with sterile glass beads for 5 min at 2000 rpm. 100 μl of the obtained lysate were incubated with approximately 50 cells of each naïve strain at 24°C. The establishment of an infection was monitored by FISH at several time points (1, 17, 48 h, 7 days, and 5–7 weeks after exposure).

### Fluorescence *in situ* hybridization

FISH was used to determine the presence and infection prevalence of *H. caryophila*, i.e., to verify the elimination of the endosymbiont after antibiotic treatment and the prevalence after experimental infection. Therefore, approximately 30 *Paramecium* cells were fixed in 2% paraformaldehyde directly on the slide (Superfrost Ultra Plus, ThermoScientific, Waltham, MA, USA) and subjected to dehydration by an ethanol gradient (10 min at 50, 80, and 100% each). Then the hybridization was carried out with probes labeled with 6-carboxyfluorescein or Cyanine 3 in hybridization buffer (900 mM l^−1^ NaCl, 20 mM l^−1^ TrisHCl, 0.01% SDS) at 46°C for 18 h. The universal probe EUB338 (5′-GCTGCCTCCCGTAGGAGT-3′; Amann et al., 1990) and the species-specific probe HoloCar1257 (5′-CCAGGTCACCCTATTGCA-3′; Castelli et al., 2015) were obtained from Eurofins Genomics (Ebersberg, Germany). After washing (900 mM l^−1^ NaCl, 20 mM l^−1^ TrisHCl, 0.01% SDS) at 48°C for 20 min to remove excess probes, the slides were air dried and mounted with Citifluor AF1 (Citifluor Ltd., London, UK). FISH results were observed by epifluorescence microscopy (Nikon Eclipse Ti, Tokio, Japan; Zeiss Axio M2, Carl Zeiss, Jena, Germany), applying different filter sets for 6-carboxyfluorescein (Nikon: EX BP450/90 nm | LP FT 500 nm | EM LP 515 nm; Zeiss: EX BP 470/40 | BS FT 495 | EM BP 525/50) and Cyanine 3 (Nikon: EX BP 510/60 nm | LP FT 560 nm | EM LP 590 nm; Zeiss: EX BP 545/25 | BS FT 570 | EM BP 605/70).

### Fitness assay

Exponential growth of the 20 used *Paramecium* lines (Table 1) was achieved by doubling the culture volume each day with diluted (1:2) bacterized CM for a period of 3 days prior to the fitness analysis. The cultivation temperature was shifted to 24°C for this adaptation phase as well as the following fitness assay. For the assay, the starting cell density was set to approximately 15 cells ml^−1^ in a total volume of 30 ml containing 20 ml of bacterized CM. Three biological replicates were included for each line. *Paramecium* cell density at each sampling point was determined by counting (three technical replicates) as described elsewhere (Krenek et al., 2011). Growth rate *r* (d^−1^) and carrying capacity *k* (cells ml^−1^) were calculated for each biological replicate by fitting a logistic growth model (Figure S1; R version 3.2.3, R Development Core Team, 2015) according to Dusi and colleagues (Dusi et al., 2014). The fitness impact caused by *H. caryophila* was assessed by comparing growth rate and carrying capacity of infected relative to endosymbiont-free lines (Figure S2; Dusi et al., 2014). The infection status of chronically and experimentally infected lines was determined by FISH at the beginning and the end of the fitness assay. Statistical significance of obtained results was tested by a two-way ANOVA (R version 3.2.3; R Development Core Team, 2015).

## Results

### Cured and experimentally infected *P. biaurelia*

**Figure 1:**
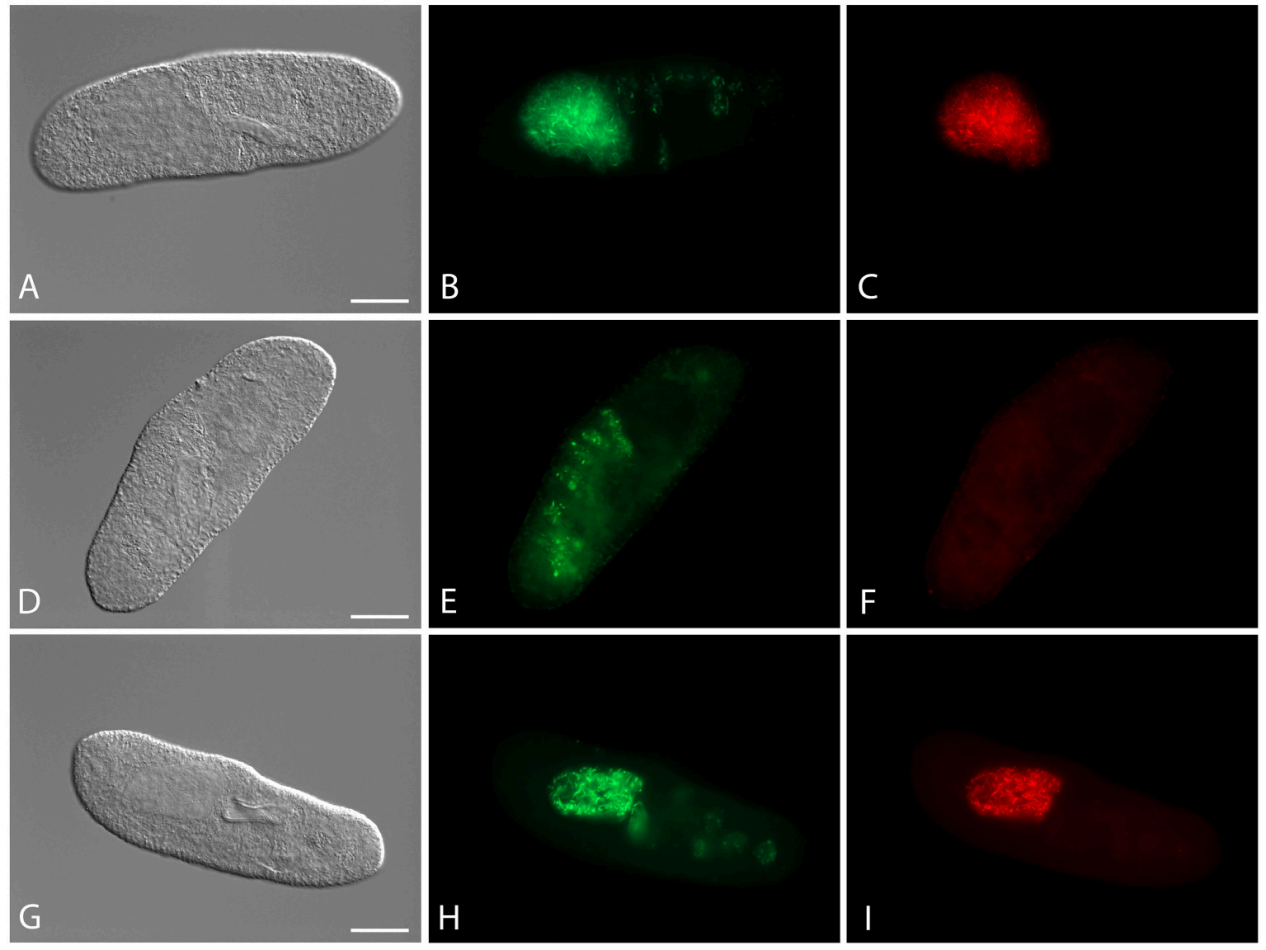
**Fluorescence *in situ* hybridization for the visualization of *Holospora caryophila* FGC3 in different *Paramecium biaurelia* lines. (A–C)** Chronically infected *P. biaurelia* FGC3_chronic; **(D–F)** *P. biaurelia* FGC3_AB, cured via antibiotic treatment from the infection; **(G–I)** experimentally infected *P. biaurelia* Ri. Used probes detect either most bacteria (EUB338; **B,E,H**) or specifically *H. caryophila* (HoloCar1257; **C,F,I)**. Bacteria present in food vacuoles show positive signals only with EUB338 **(B,E,H)**, those in the macronucleus with both probes **(B,C,H,I)**. Note that long (IF) and short (RF) rods can be observed in the infected macronuclei. Bars = 20 μm.

Four cured *Paramecium* lines (Table 1) were established through antibiotic treatment of chronically infected cells. Successful elimination of *H. caryophila* was verified repeatedly by FISH (Figure 1), the examined cells were 100% *H. caryophila*-free.

**Figure 2:**
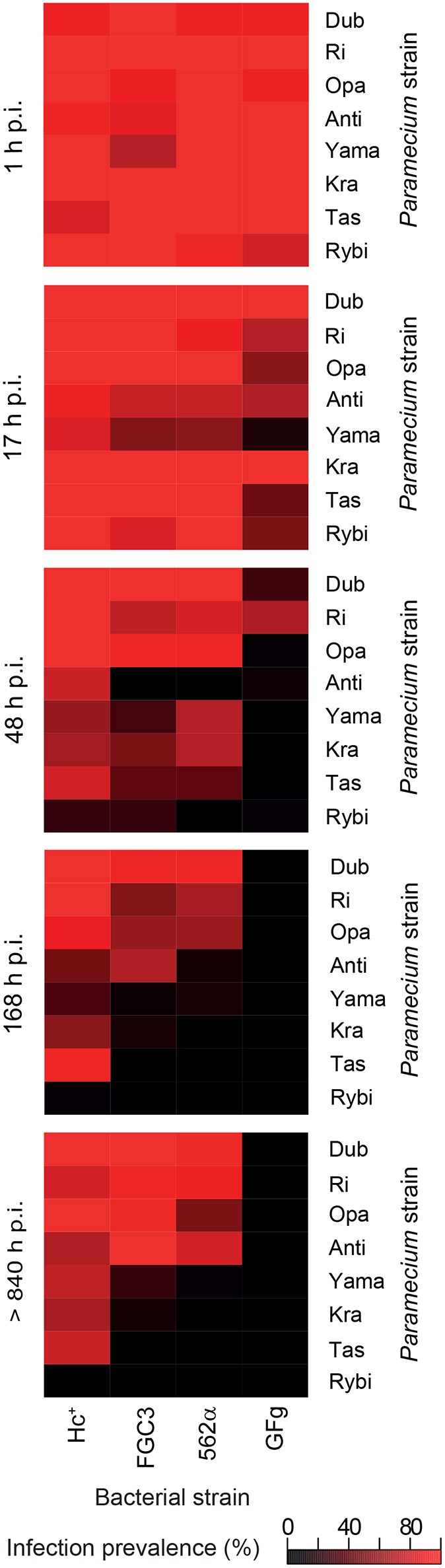
**Infection prevalence at different time points of the experimental infection experiment**. Eight naïve *Paramecium biaurelia* strains (host strain) were exposed to isolated *Holospora caryophila* belonging to four different strains. At the indicated time points after exposure (*post* infection**, p.i.) at least 20 cells of each combination were fixed and subjected to fluorescence *in situ* hybridization with probe HoloCar1257. *Paramecium* cells bearing positive signals were counted as infected regardless the subcellular localization of the bacteria (e.g., food vacuole, cytoplasm, macronucleus). Scale: percentage of infection prevalence.

The fate of the infectious bacteria in *Paramecium* was followed after exposure to *H. caryophila* (*post* infection**, p.i.) isolated from chronically infected hosts through FISH. For this experiment, a cell was considered infected when a signal with the species-specific probe HoloCar1257 was observed from food vacuoles, cytoplasm, or macronucleus (Figure 1). Depending on the bacterial strain as well as of the exposed *P. biaurelia*, rather strong differences in the bacterial infectivity respectively host susceptibility were observed (Figure 2). All experimentally infected lines showed an up-take of *H. caryophila* from the medium (Figure 2, 1 and 17 h p.i.). The initially observed overall high prevalence dropped after 48 h p.i. *Paramecium* lines exposed to *H. caryophila* GFg showed little to no positive signals henceforth. On the other hand, the majority of strains mixed with *H. caryophila* Hc^+^ (all but Rybi) maintained a high prevalence till the end of the experiment. The infection success of *H. caryophila* FGC3 and 562α remained in-between those two extremes: both were maintained in four out of eight lines.

Regarding the *Paramecium* susceptibility, half of the tested strains (Dub, Ri, Opa, and Anti) harbored an infection with any of the three *H. caryophila* strains FGC3, Hc^+^, and 562α with a prevalence higher than 68% at the end of the experiment. *P. biaurelia* Rybi was capable of ingesting *H. caryophila*, but the percentage of paramecia with intracellular bacteria strongly decreased after 17 and reached zero after 168 h p.i. for all four tested bacterial strains. Similar, *P. biaurelia* Kra and Tas harbored little or no *H. caryophila* at the late time points except for those exposed to Hc^+^.

### Fitness costs of infection

**Figure 3:**
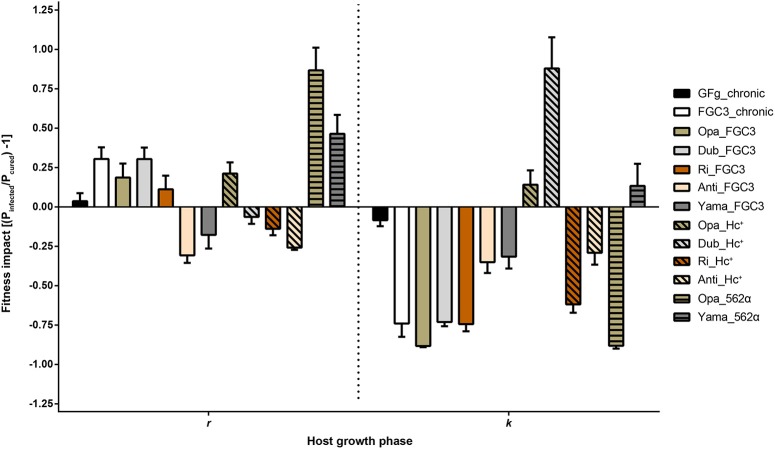
**Fitness impact on *Paramecium biaurelia* lines caused by *Holospora caryophila* during two different host growth phases**. As parameters were used the exponential growth rate (*r*) and maximal density at carrying capacity (*k*). Those values were obtained from a nonlinear parametric regression model based on the growth data obtained from three replicates. Impact was calculated as ratio of infected relative to cured *P. biaurelia* lines [(P_infected_/P_cured_) − 1] for *r* and *k*, respectively. Statistical significance of the bacterial and host strain as well as their interaction was confirmed by a two-way ANOVA.

The growth curves of 20 *P. biaurelia* lines (Table 1, Figure S3) were determined and used to obtain the fitness parameters growth rate *r* and carrying capacity *k* (Table S1). Based on these values, the relative fitness impact caused by the symbiont was calculated (Figure 3). Accordingly, host fitness was differently affected by *H. caryophila* during the exponential and stationary growth phase. The impact of the symbiont on the carrying capacity of most combinations was clearly negative. Almost all infected lines exhibited an obvious reduction in cell density compared to the matched *H. caryophila-*free line. This was especially striking in the case of *H. caryophila* FGC3 (Figure 3). Interestingly, an infection by *H. caryophila* could also resulted in a weak (Opa_Hc^+^, Yama_562α) or even strongly positive (Dub_Hc^+^) influence on the host's carrying capacity.

During the exponential growth of the host, the symbiont's effect was in most cases positive. It ranged from strongly positive (Opa_562α) to positive (Yama_562α) to weakly positive (e.g., FGC3_chronic) and even to negative effects (e.g., Dub_Hc^+^). The strain identity of *H. caryophila* and *Paramecium* and the interaction of both influenced the outcome of their interaction statistically significantly during both growth phases (Tables S2, S3).

Crossover genotype by genotype (G × G) interactions were observed (Figure S4), although not for all theoretically possible combinations fitness data were available. The interactions had effects on both, the exponential growth rate (Figure S4A) and on cell density at carrying capacity (Figure S4B). Accordingly, the specific G × G combination determined the magnitude and type (positive vs. negative) of the phenotypic response.

FISH verified the maintenance of *H. caryophila* during the fitness assay. Between 82 and 100% of the *P. biaurelia* cells representing the infected lines carried the symbiont in the macronucleus at the beginning and end of the assay. Prevalence changes within the lines over the course of the experiment were not pronounced (± 0.5–13%).

Eight lines (Hc^+^_chronic, Hc_AB, 562α_chronic, 562α_AB, Yama_Hc^+^, Anti_562α, Dub_562α, and Ri_562α) presented a much slower increase in cell number compared to all other lines (data not shown). Thus, their growth rate and carrying capacity could not be unambiguously determined and these lines were excluded from further analysis. The experimental infection of *P. biaurelia* Rybi did not result in stably infected new lines. Likewise, no new experimentally infected lines could be established with *H. caryophila* GFg.

## Discussion

Contrary to expectations, the presence of the bacterial symbionts was generally advantageous for *P. biaurelia* during exponential growth causing a higher growth rate in many lines. Bacterial virulence (a negative influence on host fitness) was mostly observed at carrying capacity. During this growth phase, the intranuclear bacteria *H. caryophila* exhibit a negative impact resulting in a decrease of the maximal reached *Paramecium* cell density for almost all combinations. These findings indicate that the bacterial impact is host growth phase dependent. Interestingly, seven *H. caryophila* × *Paramecium* combinations reveal a switch in the bacterial influence on its host from positive during exponential growth to negative during carrying capacity (Figure 3, e.g., FC3_chronic) or *vice versa* (e.g., Dub_Hc^+^). The other six combinations undergo either a stimulation (Yama_562α) or decrease (Anti_Hc^+^) during both host growth phases compared to the growth of the matching symbiont-free *Paramecium* line.

It is plausible that the amount of energy available for *Paramecium*'s division is decreased by these endosymbiotic bacteria: *H*. *caryophila* belongs to a group typically considered as energy parasites (Görtz and Schmidt, 2005). Their obligate intranuclear lifestyle requires the uptake of host resources for their own multiplication, possibly ATP via a nucleotide transporter as shown for *H. obtusa* (Linka et al., 2003). A hypothesis of how *H. caryophila* positively affect their hosts' growth rate is harder to come by. Several studies revealed that *Paramecium* infected with *Holospora* can obtain benefits from the infection. For example, infection with *H. undulata* provides an increased resistance to osmotic stress (Duncan et al., 2010). *H. obtusa* increases *P. caudatum* tolerance to heat-shock and contributes to maintain the ciliary movement of the host even at temperatures above and below the normal physiological temperature range of the host (Hori and Fujishima, 2003; Fujishima et al., 2005). Likely, the underlying molecular mechanism involves an increase of *Paramecium* heat-shock gene (*hsp*) expression caused by the symbiont (Hori and Fujishima, 2003). Enhanced *hsp* mRNA levels have also been observed for *Paramecium bursaria* living in symbiosis with the photosynthetic microalgae *Chlorella* (Kodama et al., 2014). If *H. caryophila* likewise effects the *hsp* expression of its host has not yet been studied. How such an elevated Hsp70 level could result in an increased host growth rate or which other factors might be involved remain open questions for further investigations.

*H. caryophila* is capable of both vertical and horizontal transmission accomplished by the respective morphological cell form. The reproductive form is essential for vertical transmission of the bacteria when the host cell divides. Horizontal transmission via uptake from the medium is connected with the infectious form of *H. caryophila* (Görtz and Schmidt, 2005). In this study, the latter was prerequisite for the generation of experimentally infected lines (Figure 2). For the fitness assay, all chosen infected lines manifested a prevalence of *H. caryophila* of 82% or higher. Thus, in this experiment the amount of symbiont-free *Paramecium* cells available for horizontal infection can be neglected. A similar scenario (the majority of paramecia carrying an infection) might be assumed for natural populations with a long-term infection by *H. caryophila*. When nutrient availability and space are not limited and the host is in the exponential growth phase, the obligate endosymbionts optimize their own transmission success via vertical transmission distinctly when they exhibit a positive influence on the division rate of their host. This results in a higher number of infected individuals. During the stationary phase, the host population reaches the maximal cell density with a constant cell number caused by an equilibrium of dividing and dying cells. An increase of the total bacterial population by means of vertical transmission is not possible any longer. In the fitness assay, we observe a negative impact of *H. caryophila* in this phase for many combinations. This might be caused by a stronger exploitation of host resources than during exponential growth and/or the withdrawal of host stimulation which results in an increased virulence of the bacteria. Presumably, this switch from a positive to a negative impact is synchronized with the beginning differentiation of *H. caryophila* from reproductive to infectious form (which is capable of horizontal transmission) and consequently the spread of the obligate endosymbionts. Accordingly, the two biochemically and morphologically different forms of *H. caryophila* might independently enhance or decrease the host's division rate by so-far unknown mechanisms. Additional experiments should be conducted at the transition from exponential to stationary host growth with the aim to detect (and possibly enumerate) the amount of both *H. caryophila* forms. This would provide a better understanding of the effect of each form on the host population.

Infection success (Figure 2) and fitness impact (Figure 3, Figure S4) of the tested endosymbionts on different *P. biaurelia* lines were not only dependent on either strain but were specific for the respective G × G combination. In many host-parasite associations the specific interaction of both genotypes determines the outcome of the infection (Lambrechts et al., 2006, 2009). Our study shows that the importance of the G × G interaction is not limited to early steps of infection or the establishment of a stable association but also influences the fitness of the symbiotic partner in different growth phases.

All naturally infected strains used in this study were originally isolated in Germany or Italy (Table 1). In a recent review of the distribution of *Holospora* species including *H. caryophila* (Serra et al., 2016), the authors list the countries with positive reports: Austria, Czech Republic, Germany, Italy, Russia, Ukraine, and USA. The here employed naïve *P. biaurelia* strains derive from different European regions as well as countries in Asia and Australia (Table 1), thus they do not overlap with the known incidence of *H. caryophila* (with the exception of Russia). During the infection experiment, strains from Russia (Anti, Dub) and Europe (Ri, Opa) were most susceptible whereas more resistant strains (Yama, Tas) originate from Asia and Australia (Figure 2). However, also strains from Poland (Kra) and Russia (Rybi) belong to the resistant group. Although, it might be tempting to formulate hypotheses based on these geographic patterns, our knowledge of the natural distribution of symbiotic bacteria of protists including *Holospora* is scarce. *H. caryophila*'s overrepresentation in Europe is most likely the result of a sampling bias. Only an expansion of previous efforts will enable us to unravel the diversity and distribution of bacterial symbionts of unicellular hosts.

## Author contributions

MS designed the study; CB, LK, and KG performed the experiments; data analysis was carried out by CB, LK, KG, and MS; LK and MS prepared graphs and figures; TB and GP contributed to the interpretation and discussion of results; CB, LK, KG, and MS conducted the literature review, MS wrote the manuscript with contributions of CB and LK. All authors read and approved the final manuscript.

## Funding

This research was supported by the Volkswagen Foundation (project number: 84816), the Seventh Framework Programme IRSES of the European Commission (CINAR-PATHOBACTER 247658), the European Cooperation in Science and Technology BMBS COST Action BM1102, and by the Ministero dell'Istruzione, dell'Università e della Ricerca (project protocol: 2012A4F828 002). The article processing charge was funded by the German Research Foundation (DFG) and the Albert Ludwigs University Freiburg in the funding programme Open Access Publishing.

### Conflict of interest statement

The authors declare that the research was conducted in the absence of any commercial or financial relationships that could be construed as a potential conflict of interest.
