# Supplementary material for: Fitness Impact of Obligate Intranuclear Bacterial Symbionts Depends on Host Growth Phase
Source: Front Microbiol. 2016 Dec 22;7:2084. doi: 10.3389/fmicb.2016.02084 (PMC5177645; doi:10.3389/fmicb.2016.02084)
Supplement: Supplementary file 1 [file Table1.docx]

Supplementary Material

# Fitness Impact of Obligate Intranuclear Bacterial Symbionts Depends on Host Growth Phase

**Chiara Bella^1,2,†^, Lars Koehler^1,3, †^, Katrin Grosser^1,3^, Thomas U. Berendonk^3^, Giulio Petroni^2^, Martina Schrallhammer^1,3,*^**

^*^ **Correspondence**: Martina Schrallhammer, [martina.schrallhammer@biologie.uni-freiburg.de](mailto:martina.schrallhammer@biologie.uni-freiburg.de)

**Supplementary Table S1: Fitness parameters exponential growth rate *r* and carrying capacity *k* of *Paramecium biaurelia*.**

| ***P. biaurelia* line** | **Infection status** | ***r* ± SD (h^-1^)** | ***k* ± SD (cells ml^-1^)** |
| --- | --- | --- | --- |
| FGC3_AB | Cured | 0.0557 ± 0.0042 | 1310 ± 72 |
| FGC3_chronic | FGC3 | 0.0725 ± 0.0036 | 339 ± 174 |
| GFg_AB^a^ | Cured | 0.0717 ± 0.0036 | 2432 ± 125 |
| GFg_chronic^a^ | GFg | 0.0743 ± 0.0043 | 2139 ± 87 |
| Anti | Naïve | 0.0628 ± 0.0017 | 214 ± 27 |
| Anti_Hc^+^ | Hc^+^ | 0.0466 ± 0.0006 | 151 ± 15 |
| Anti_FGC3 | FGC3 | 0.0435 ± 0.0046 | 138 ± 14 |
| Ri | Naïve | 0.0621 ± 0.0011 | 340 ± 62 |
| Ri_Hc^+^ | Hc^+^ | 0.0535 ± 0.0039 | 128 ± 10 |
| Ri_FGC3 | FGC3 | 0.0690 ± 0.0085 | 86 ± 17 |
| Dub | Naïve | 0.0481 ± 0.0035 | 356 ± 61 |
| Dub_Hc^+^ | Hc^+^ | 0.0514 ± 0.0011 | 663 ± 33 |
| Dub_FGC3 | FGC3 | 0.0670 ± 0.0058 | 95 ± 2 |
| Yama | Naïve | 0.0600 ± 0.0047 | 1024 ± 180 |
| Yama_FGC3 | FGC3 | 0.0493 ± 0.0072 | 694 ± 23 |
| Yama_562α | 562α | 0.0877 ± 0.0088 | 1149 ± 112 |
| Opa | Naïve | 0.0517 ± 0.0046 | 672 ± 73 |
| Opa_Hc^+^ | Hc^+^ | 0.0624 ± 0.0005 | 764 ± 57 |
| Opa_FGC3 | FGC3 | 0.0611 ± 0.0045 | 78 ± 2 |
| Opa_562α | 562α | 0.0962 ± 0.0077 | 79 ± 17 |

^a^ The host does not belong to the species *Paramecium biaurelia,* but to the closely related *Paramecium octaurelia.*

Values represent mean of three replicates ± SD; cured – the symbionts were removed by antibiotic treatment; naïve – no infection has been observed in this strain since its isolation from the environment.
