# Supplementary material for: Fitness Impact of Obligate Intranuclear Bacterial Symbionts Depends on Host Growth Phase
Source: Front Microbiol. 2016 Dec 22;7:2084. doi: 10.3389/fmicb.2016.02084 (PMC5177645; doi:10.3389/fmicb.2016.02084)
Supplement: Supplementary file 2 [file Table2.docx]

Supplementary Material

# Fitness Impact of Obligate Intranuclear Bacterial Symbionts Depends on Host Growth Phase

**Chiara Bella^1,2,†^, Lars Koehler^1,3, †^, Katrin Grosser^1,3^, Thomas U. Berendonk^3^, Giulio Petroni^2^, Martina Schrallhammer^1,3,*^**

^*^ **Correspondence**: Martina Schrallhammer, [martina.schrallhammer@biologie.uni-freiburg.de](mailto:martina.schrallhammer@biologie.uni-freiburg.de)

**Supplementary Table S2: Two-way ANOVA summary of the impact of *H. caryophila* on the fitness parameter exponential growth rate *r* of *Paramecium biaurelia*.**

|  | **Df** | **Sum Sq** | **Mean Sq** | **F value** | **P value** |
| --- | --- | --- | --- | --- | --- |
| Host strain | 6 | 4.0531 | 0.67552 | 105.99 | < 2e-16 *** |
| Bacterial strain | 2 | 3.2400 | 1.62001 | 254.18 | < 2e-16 *** |
| Host:bacteria | 4 | 0.3837 | 0.09592 | 15.05 | 9.3e-09 *** |
| Residuals | 65 | 0.4143 | 0.00637 |  |  |
