# Supplementary material for: Fitness Impact of Obligate Intranuclear Bacterial Symbionts Depends on Host Growth Phase
Source: Front Microbiol. 2016 Dec 22;7:2084. doi: 10.3389/fmicb.2016.02084 (PMC5177645; doi:10.3389/fmicb.2016.02084)
Supplement: Supplementary file 4 [file Image1.pdf]

Supplementary Material
Fitness Impact of Obligate Intranuclear Bacterial Symbionts Depends on Host Growth Phase

Chiara Bella[1,2,†], Lars Koehler[1,3, †], Katrin Grosser[1,3], Thomas U. Berendonk[3], Giulio Petroni[2], Martina Schrallhammer[1,3,*]

[*] **Correspondence**: Martina Schrallhammer, martina.schrallhammer@biologie.uni-freiburg.de

Supplementary Equation S1: Logistic growth model

$$y_t = \frac{(k * y_0)}{y_0 + (k - y_0) * e^{-r*t}}$$

y_0 represents the initial host concentration (cells ml^{-1}), y_t the host density (cells ml^{-1}) after time t (d), r the exponential growth rate, and k the carrying capacity
