# Supplementary material for: Fitness Impact of Obligate Intranuclear Bacterial Symbionts Depends on Host Growth Phase
Source: Front Microbiol. 2016 Dec 22;7:2084. doi: 10.3389/fmicb.2016.02084 (PMC5177645; doi:10.3389/fmicb.2016.02084)
Supplement: Supplementary file 5 [file Image2.pdf]

Supplementary Material
Fitness Impact of Obligate Intranuclear Bacterial Symbionts Depends on Host Growth Phase

Chiara Bella[1,2,†], Lars Koehler[1,3,†], Katrin Grosser[1,3], Thomas U. Berendonk[3], Giulio Petroni[2], Martina Schrallhammer[1,3,*]

[*] **Correspondence**: Martina Schrallhammer, martina.schrallhammer@biologie.uni-freiburg.de

Supplementary Equation S2: Determination of the fitness impact of infection.

$$fitness\ impact = \left(\frac{P_{infected}}{P_{not\ infected}}\right) - 1$$

$P_{infected}$ represents the *r* respectively *k* values of paramecia carrying *Holospora caryophila*; $P_{not\ infected}$ of symbiont-free paramecia
