# Supplementary material for: Fitness Impact of Obligate Intranuclear Bacterial Symbionts Depends on Host Growth Phase
Source: Front Microbiol. 2016 Dec 22;7:2084. doi: 10.3389/fmicb.2016.02084 (PMC5177645; doi:10.3389/fmicb.2016.02084)
Supplement: Supplementary file 6 [file Image3.pdf]

Supplementary Material
Fitness Impact of Obligate Intranuclear Bacterial Symbionts Depends on Host Growth Phase

Chiara Bella[1,2,†], Lars Koehler[1,3, †], Katrin Grosser[1,3], Thomas U. Berendonk[3], Giulio Petroni[2], Martina Schrallhammer[1,3,*]

[*] **Correspondence**: Martina Schrallhammer, martina.schrallhammer@biologie.uni-freiburg.de

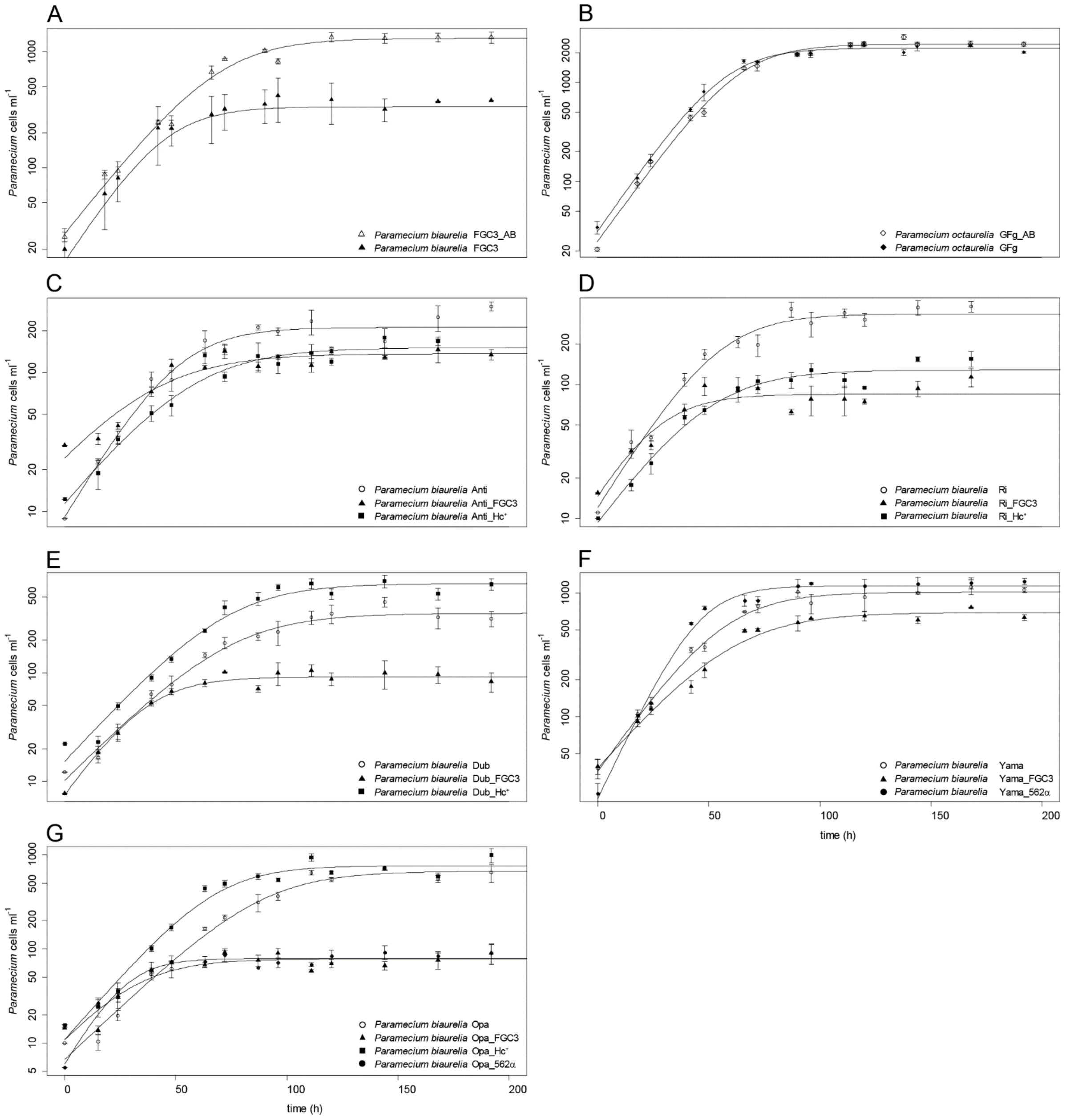

Figure S1: Growth curves of *Paramecium biaurelia* lines. Data points represent the mean cell density of three replicates ± SD at different time points (in hours). The regressions (lines) were fitted by a nonlinear parametric regression model. Filled symbols indicated lines infected by *H. caryophila* FGC3 (triangle; **A, C-G**), GFg (diamond; **B**), Hc^+ (square; **C-E, G**), or 562α (circle; **F, G**), respectively. Empty symbols indicate naïve (empty circle; **C-G**) or cured (via antibiotics, AB) lines (empty triangle or diamond; **A, B**).
