# Supplementary material for: Fitness Impact of Obligate Intranuclear Bacterial Symbionts Depends on Host Growth Phase
Source: Front Microbiol. 2016 Dec 22;7:2084. doi: 10.3389/fmicb.2016.02084 (PMC5177645; doi:10.3389/fmicb.2016.02084)
Supplement: Supplementary file 7 [file Image4.pdf]

Supplementary Material

Fitness Impact of Obligate Intranuclear Bacterial Symbionts Depends on Host Growth Phase

Chiara Bella[1,2,†], Lars Koehler[1,3, †], Katrin Grosser[1,3], Thomas U. Berendonk[3], Giulio Petroni[2], Martina Schrallhammer[1,3,*]

[*] **Correspondence**: Martina Schrallhammer, martina.schrallhammer@biologie.uni-freiburg.de

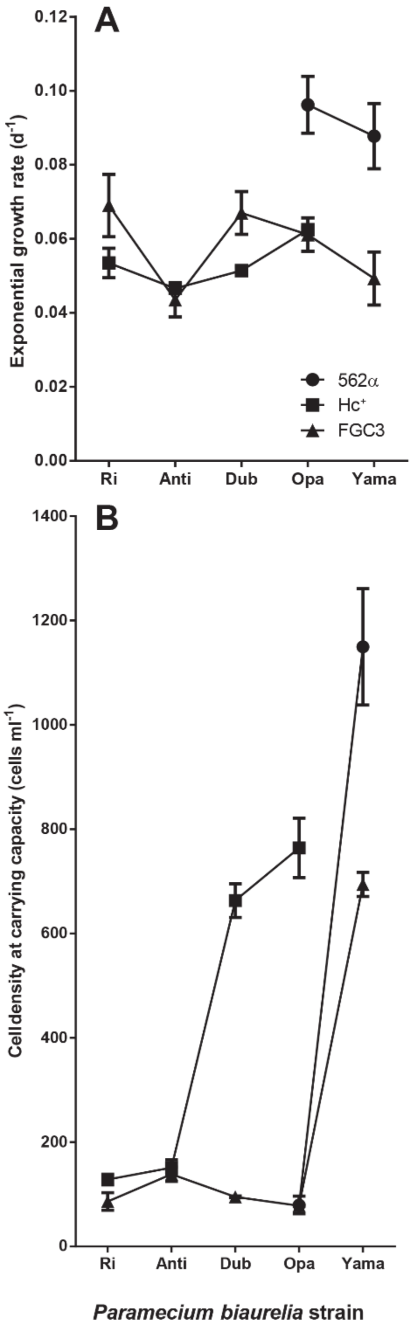

Figure S2: Graphic representation of interaction of *Paramecium biaurelia* strains and different *Holospora caryophila* symbionts. Interaction during exponential growth (**A**) and at carrying capacity (**B**) of the host. *P. biaurelia* strains are indicated on the x-axis, bacterial strains are represented by symbols: *Holospora caryophila* 562α (circle), Hc^+ (square), and FGC3 (triangle). Crossing lines give an indication of genotype by genotype interactions. Each data point represents the mean of three replicates ± SD, lines connect points representing the same bacterial strain.
